# Better learning in schools to improve attitudes toward abstinence and intentions for safer sex among adolescents in urban Nepal

**DOI:** 10.1186/1471-2458-13-244

**Published:** 2013-03-20

**Authors:** Rachana Manandhar Shrestha, Keiko Otsuka, Krishna C Poudel, Junko Yasuoka, Medin Lamichhane, Masamine Jimba

**Affiliations:** 1 Department of Community and Global Health, Graduate School of Medicine, The University of Tokyo, 7-3-1, Hongo, Bunkyo-ku, Tokyo 113-0033, Japan; 2 Department of Public Health, School of Public Health and Health Sciences, University of Massachusetts Amherst, 316 Arnold House, 715 North Pleasant St, Amherst, MA 01003-9304, USA; 3 Ullens School, GPO Box. 8975, Kathmandu, EPC 1477, Nepal

**Keywords:** Students, School health services, Sex education, Attitudes, Intentions, Abstinence, Safer sex, Nepal

## Abstract

**Background:**

School-based sex education is an effective medium to convey health information and skills about preventing sexually transmitted infections (STIs) and unwanted pregnancies among adolescents. However, research on school-based sex education is limited in many developing countries, including Nepal. This study thus had two main objectives: (1) to assess students’ evaluation of school-based sex education, and (2) to examine the associations between students’ evaluations of school-based sex education and their (a) attitudes toward abstinence and (b) intentions for safer sex.

**Methods:**

This cross-sectional study was conducted among 634 students from six schools in the Kathmandu Valley during May–June 2010. We used a self-administered questionnaire to assess students’ evaluations of school-based sex education, attitudes toward abstinence, and intentions for safer sex. The data were then analyzed using multiple linear regression models.

**Results:**

Regarding “information on HIV and sexual health”, many students perceived that they received the least amount of information on HIV counseling and testing centers (mean 2.29, SD 1.00) through their schools. In terms of “support and involvement of teachers and parents” in sex education, parents’ participation ranked as the lowest (mean 1.81, SD 1.01). Audiotapes were reported as the least used among the listed “teaching aids for sexual health education” (mean 1.54, SD 0.82). In multivariate analysis, receiving more “information on HIV and sexual health” was positively associated with more positive “attitudes toward abstinence” (β = 0.11, p = <0.018) and greater “intentions for safer sex” (β = 0.17, p = <0.001) among students. Similarly, increased “support and involvement from teachers and parents” was also positively associated with more positive “attitudes toward abstinence” (β = 0.16, p = <0.001) and greater “intentions for safer sex” (β = 0.15, p = <0.002).

**Conclusion:**

Our results suggest that students’ needs and expectations regarding HIV and sexual health education are not being met through their schools. Moreover, comprehensive information on HIV and sexual health along with increased support and involvement of teachers and parents in sex education might help to improve adolescents’ attitudes toward abstinence and intentions for safer sex. Adapting future school-based interventions to incorporate such elements may thus be an effective strategy to promote adolescent sexual health.

## Background

School-based sex education is a promising medium for reaching many adolescents with fundamental health information and life skills that can prevent unintended pregnancies [1] and sexually transmitted infections (STIs) including HIV/AIDS [1-4]. A review of 83 sex and HIV education programs revealed that such programs can be effective in improving sexual behaviors among youth, both in developed and developing countries [5]. These programs are effective in increasing knowledge, changing attitudes [6], delaying the first sexual experience [5,7], reducing the frequency of sexual intercourse and the number of sexual partners [7-10], lowering pregnancy rates [1], and increasing condom or contraceptive use [1,5,7] among young people.

Adolescents’ premarital sexual activities are increasing in the countries around the world, many of which are risky, unplanned [11-13], and unprotected [11,12,14,15]. Among Nepalese adolescents too, premarital sexual activities are on the rise [12,16,17]. Such activities make adolescents one of the most vulnerable groups for HIV infection throughout the world and Nepal is no exception [11,18,19]. Therefore, this group needs serious and timely attention and active intervention.

The government of Nepal has designed a school curriculum which provides basic education on adolescence, sexual and reproductive health for all students from grades six through ten [20]. School-based sex education has the potential to improve adolescents’ sexual health knowledge [21], attitudes, and intentions in ways that can affect their sexual behaviors [5]. Specifically, positive attitudes and intentions regarding sexual behaviors act as mediating or motivating factors contributing to positive sexual behaviors [5,22]. Since school-based sex education can influence students’ knowledge, beliefs, and intentions regarding sexual health, it is important to fully explicate and address the social and cultural challenges of school-based sex education.

School-based sex education remains a challenge, particularly in developing countries like Nepal. It is severely constrained by social and cultural taboos on discussing sex, STIs, and HIV/AIDS at school [23,24]. Most of the schools often entirely lack student-friendly and positive environment, and a formal guidance or counseling system for students to seek advice and support on sexual health issues [23-25]. Because the information students need and want to receive is often incomplete and insufficient, they are frequently dissatisfied with the quality of sex education provided in their schools [23,24,26].

Research demonstrates that teachers are often not properly trained and lack the necessary skills to teach sexual health topics [23,25]. Students from India, Kenya, Nepal, and Korea alike felt that their teachers were unable to teach sexual health topics effectively, sometimes even simply being left idle during sexual health lessons [23,24,26]. From the teachers’ perspectives in Nepal, India, and Kenya, support was lacking from school principals, parents, and the community for educating students on sexual health issues [23,24]. Overall, both teachers and students remarked on the lack of training, teaching aids, interactive teaching methods, and on the inadequate amount of time allocated for sex education [23,24,26].

Parents’ involvement and support can improve the effectiveness of health education programs [27], especially in schools that are providing sex education [25]. Communication with parents and active parental participation in educating adolescents about sexual health issues can demonstrably influence adolescents’ attitudes and beliefs [25], and may also help to delay the onset of sexual relations [28] and promote safer sexual behaviors [29,30]. However, many Nepalese parents have especially low levels of awareness on matters regarding their children’s education, and thus are often unaware of the conduct or content of sex education in the schools [31]. They are also typically afraid that sex education may encourage their children to engage in sexual activity at an early age [25]. Thus, it is difficult for parents and adolescents to discuss sexual health issues [28]. Other common obstacles to effective school-based sex education are the poor quality of textbooks, the lack of teaching materials and resources, and insufficient time allocated for sexual health lessons [4,17,24,32].

Despite substantial positive evidence on the effectiveness of school-based sex education, only a handful of studies are available on school-based sex education programs from South Asian countries. A few qualitative and review studies have explored the difficulties of providing school-based sex education in Nepal [23,25,31], but quantitative evaluation on school-based sex education is still lacking. Therefore, the association between school-based sex education and student attitudes and intentions regarding sexual behavior remains unknown. Our study thus aimed, first of all, to assess the students’ evaluation of school-based sex education. Along the same lines, the study’s second objective was to examine the associations between students’ evaluations of school-based sex education and students’ (a) attitudes toward abstinence and (b) intentions for safer sex. We hypothesized that those students who evaluated their school-based sex education experience as poor might have poorer attitudes toward abstinence and intentions for safer sex.

## Methods

### Study design and sampling

We conducted this cross-sectional study in the Kathmandu Valley. As the topic of sexuality is rather sensitive in the Nepalese context, we identified the target schools through convenience sampling techniques. Through personal connections, we communicated initially with a single school in the Valley. Using this school’s extensive network of connections with other schools in the region, we then recruited further schools for the evaluation.

We used Power and Precision software, version 4 (Biostat, Englewood, NJ, USA) to calculate the minimum sample size required for 80% power, with level of significance set at 5% for a 95% confidence interval. Considering the sensitivity of the topic and the sample size required for effective factor analysis [33], we extrapolated the calculated 360 minimum sample size to more than 500. After collecting data from 703 students from six schools, we stopped recruiting further schools and students.

### Participants

We recruited all the students studying in the 9th and 10th grades from six different schools in the Kathmandu Valley. Students attending these schools commute from all three districts of the Kathmandu Valley. Moreover, all sampled schools follow the Nepalese government curriculum, which includes basic education on adolescence, sexual and reproductive health for students in grades six through ten, and we confirmed with school administrators that students had already covered the pertinent textbook chapters prior to data collection. All students studying in the 9th and 10th grades and who returned the distributed informed consent sheet were eligible for participation in the study. Any students who did not return the informed consent form with their parent’s signature or who did not freely consent to participate were excluded from the study.

### Instrument development

We developed a preliminary version of the questionnaire (Additional file 1) in English based on similar previous studies [24,34-37]. The questionnaire was translated into Nepali and then back translated into English by different bilingual individuals. Each of the translators had at least a master’s degree in health science. We compared the original and back-translated versions to assure quality and consistency. We then pre-tested the Nepali- language questionnaire among 70 students prior to the final data collection. Questionnaire content was also discussed with 4 public health researchers, 1 scale developer, 3 schoolteachers, 4 principals, and 10 students to verify the relevance of the items in the Nepalese context. Necessary modifications in wording and item order were made based on pre-test results and discussion feedbacks.

### Variables and measurements

Study variables and questionnaire items were selected based on review of the existing literature. We then developed the framework for the school-related factors and variables that might be associated with students’ attitudes toward abstinence and intentions for safer sex. Finally, we developed the questionnaire to measure (a) socio-demographic characteristics, (b) students’ evaluations of school-based sex education, (c) students’ attitudes toward abstinence, and (d) students’ intentions for safer sex.

#### Socio-demographic characteristics

We collected information on students’ age, gender, school grade, school number (out of six schools participating in the study), living arrangements (living with both parents, a single parent, or other), and the education levels of their fathers and mothers. We categorized paternal and maternal education into illiterate, lower secondary level or below (up to eight years of formal education), and secondary level and above (more than eight years of formal education).

#### Students’ evaluations of school-based sex education

We assessed students’ evaluations of school-based sex education through 36 items adapted from previous studies and reports [24,34,35]. A principal component analysis was conducted on 35 items to deduce three factors and determine the factor structure of these items [33]. We designated the three factors thus identified as (a) information on HIV and sexual health, (b) support and involvement of teachers and parents, and (c) teaching aids for school-based sex education. One further item was on time allocated for sex education.

(1) Information on HIV and sexual health

The factor “Information on HIV and sexual health” contained 21 items covering different types of information on HIV and sexual health. For each item, we also asked students about the extent to which they had learned such information from their schools. Responses were recorded on a 4-point Likert scale ranging from 1 (*not at all)* to 4 (*completely*). Finally, we calculated the total score for this factor by summing all 21 items, scores ranging from 21 to 84. A higher total score indicated that the student had received a greater level of information on HIV and sexual health from his or her school. Cronbach’s alpha for this factor was 0.92 in our study.

(2) Support and involvement of teachers and parents

The factor “Support and involvement of teachers and parents” comprised 8 items covering students’ perceptions of teachers’ knowledge and skills and of the support and involvement of their parents regarding sexual health issues. Responses were reported on a 4-point Likert scale ranging from 1 (*not at all*) to 4 (*completely*). The total score was then calculated as the sum of all 8 items, ranging from 8 to 32. Higher total scores indicated that students believed that their teachers and parents were more involved and/or supportive in conveying information about sexual health issues. Cronbach’s alpha for this factor was 0.81 in our study.

(3) Teaching aids for school-based sex education

The factor “Teaching aids for school-based sex education” contained 6 items asking whether certain teaching aids were used to teach sex education in the students’ schools. Responses for these items were recorded on a 4-point Likert scale ranging from 1 (*not at all*) to 4 (*completely*). The total score, calculated as the sum of the 6 items, ranged from 6 to 24, with higher scores indicating that students believed teaching aids were more extensively used to teach sexual health topics in their schools. Cronbach’s alpha for this factor was 0.74 in our study.

(4) Time allocated for school-based sex education

We assessed the “time allocated for sex education” using a single item. Responses were on a 4-point Likert scale ranging from 1 (*not at all*) to 4 (*completely*), with higher score meaning that the student believed a greater amount of time was allocated for sex education.

#### Attitudes toward abstinence

We assessed students’ attitudes toward abstinence using an “attitudes toward abstinence” scale adapted from a previous study conducted in Northern Utah in the United States [36]. The scale consisted of a set of 12 items measuring the students’ values about the appropriateness of adolescent sexual intercourse. Students responded to statements like “It is important for me to not have sexual intercourse before I get married” and “Even if I am physically mature, that doesn’t mean I’m ready to have sex.” We reverse-coded negative statements such as “It is all right for teenagers to have sexual intercourse before they are married if they are in love” during the data analysis process. Responses were recorded on a 5-point Likert scale ranging from 1 (*strongly disagree*) to 5 (*strongly agree*). Total summed scores for the scale ranged from 12 to 60, with higher scores indicating a more positive attitudes toward abstinence. Cronbach’s alpha for the scale was 0.68 in this study.

#### Intentions for safer sex

We assessed student intentions for safer sex using a 6-item “intentions for safer sex” scale adapted from a previous study [37]. This scale was originally developed in the United States to predict safer sex intentions among juvenile delinquents aged 13–18 years. The scale included items such as “I will make sure a condom is used when I have sex” and “I will only have one sexual relationship at a time.” Students responded to these items on a 4-point Likert scale ranging from 1 (Agree) to 4 (Disagree). We reverse-coded all items and summed all scale items to yield total scores ranging from 6 to 24, with higher scores reflecting greater intentions for safer sex. Cronbach’s alpha for the scale was 0.61 in this study.

### Data collection

We collected data from May to July 2010. Students filled out the self-administered Nepali-language questionnaire during regular class hours, a process lasting about 40–45 minutes. They answered the questionnaire anonymously in their teachers’ absence and, after completion, returned it in a sealed envelope to the researcher. The first author provided clear instructions to the students before data collection and was available throughout to answer any queries.

### Statistical analysis

Outliers and incomplete questionnaires were removed during quality check of the entered data. Initially, we calculated descriptive statistics for the 36 items individually to assess students’ evaluation of school-based sex education. Specifically, we calculated the mean for scores on each item and ranked them in order of lowest to highest. Lower mean scores indicated that students believed that the particular item was addressed poorly or was insufficient in their school and vice versa.

Bivariate regression analyses were then conducted to assess the unadjusted associations. In conducting bivariate and multivariate analyses, we first calculated the total scores for each of the factors and scales used in this study. We then assessed multicollinearity for each model; variance inflation factors (VIFs) for all the predictor variables were less than 3.0. Finally, we introduced all the variables into multiple linear regression models to determine the adjusted associations between students’ evaluations of school-based sex education and student’s (a) attitudes toward abstinence and (b) intentions for safer sex. We assessed the fit of the model based on the F-statistic of the test. The level of significance was set at p < 0.05 for all statistical tests. All analyses were conducted using SPSS version 16.0 for Windows (SPSS Inc., Chicago, IL, USA).

### Ethical considerations

We distributed the informed consent form and information sheets to students one day prior to the data collection. They then returned the informed consent form with their parent’s signature to participate voluntarily in the study. All students were assured that their participation was voluntary and that they could refuse to respond to any or all of the questionnaire items at any time. We managed the collected data with confidentiality. The ethical committees of the University of Tokyo and the Nepal Health Research Council (NHRC) reviewed and approved the study protocol.

## Results

We approached 789 students in six schools, of whom 703 students voluntarily participated in the study; 86 (10.9%) were excluded because they did not return the signed informed consent or declined to participate. We further removed 17 outliers and 52 incomplete questionnaires out of the data obtained from the 703 surveyed students. In the end, we thus included data from 634 students in the analysis.

### Characteristics of the participants

Of the 634 participants shown in Table 1, there were almost equal numbers of male (49.2%) and female (50.8%) students. The mean age was 15.1 (SD 1.3) years. Overall, 52.5% of students were in the 9th grade and 47.5% were in the 10th grade. Most of the students (70.3%) lived with both parents. Regarding parental education levels, a majority of fathers (56.8%) and mothers (59.5%) alike had an education level up to the lower secondary level (eight years of formal education). Most of the students (98.1%) responded that they had experienced sexual health education lessons at one point in their schooling, and 96.2% had begun these lessons at the lower secondary level (i.e., at six to eight years of formal education).

**Table 1:** Characteristics of the participants

**Variables**		
**Age (mean, SD)**	15.12	1.25
	**n**	**%**
**Gender**		
Male	312	49.21
Female	322	50.79
**Grade**		
9	333	52.52
10	301	47.48
**School**		
School 1	136	21.45
School 2	116	18.30
School 3	82	12.93
School 4	136	21.45
School 5	62	9.78
School 6	102	16.09
**Living arrangements**		
Both parents	446	70.35
Single parent	63	9.94
Other	125	19.71
**Father’s education**		
Illiterate	205	32.54
≤Lower secondary level	358	56.83
≥Secondary level	67	10.63
**Mother’s education**		
Illiterate	72	11.46
≤Lower secondary level	374	59.56
≥Secondary level	182	28.98
**Previously received sexual health education lessons in school**		
Yes	622	98.11
No	12	1.89
**Level at which sexual health education began**		
Primary level	5	0.79
Lower secondary level	610	96.21
Secondary level	19	3.00

### Students’ evaluations of school-based sex education

Table 2 shows students’ evaluations of school-based sex education according to three different factors: (a) information on HIV and sexual health, as measured by 21 items, (b) support and involvement of teachers and parents, as measured by 8 items, and (c) teaching aids for school-based sex education, as measured by 6 items. One further item covered the amount of time allocated for school-based sex education.

**Table 2:** Students’ evaluations of school-based sex education

**Have you learned about the following topics in your school’s sexual health education lessons? (Information on HIV and sexual health)**	**Mean**	**SD**
Where HIV counseling and testing can be received	2.29	1.00
Relationships with opposite sex	2.58	1.07
Communication with parents or other trusted adults about sexual health topics	2.53	1.04
Contraceptive use, effectiveness, and how they work	2.60	1.02
Consequences of unintended pregnancy	2.62	1.08
Number of sexual partners	2.66	1.03
Local sources for obtaining condoms and other contraceptives	2.70	1.03
Perception of peer norms about sex and perception of peer sexual behavior	2.70	1.07
HIV counseling and testing	2.71	1.00
Abstinence	2.75	1.01
Frequency of sex	2.77	1.00
Probability of becoming pregnant or causing a pregnancy if sexually active	2.78	1.02
STI testing and treatment	3.00	0.99
Emotional changes that occur in boys and girls during adolescence	3.09	0.91
Self efficacy to refuse sex and to use condoms during sexual intercourse	3.09	0.96
Condom use	3.13	1.04
Symptoms of HIV/AIDS and STIs	3.15	0.90
Modes of HIV/AIDS and STI transmission	3.17	0.90
Consequences of HIV/AIDS and STIs	3.20	0.87
Physical changes that occur in boys and girls during adolescence	3.30	0.87
Susceptibility to contracting HIV/AIDS and STIs	3.33	0.85
**Do you believe in the following about your teachers and parents/guardian? (Support and involvement of teachers and parents)**		
My parents/guardian participate in sexual health education	1.83	1.01
My parents/guardian are aware about sexual health education in my school	2.40	1.01
The Principal is committed to have sexual health education taught in this school	2.66	1.15
Teachers in this school take teaching sexual health education seriously	2.71	1.04
Teachers in this school have sufficient skills to teach sexual health education	2.77	0.94
My parents/guardian support sexual health education in my school	2.79	1.03
Teachers in this school are happy to teach sexual health education	2.93	1.00
Teachers in this school have enough knowledge to teach sexual health	2.95	0.97
**Do you think that your school used following teaching materials and resources in its sexual health education lessons? (Teaching aids for sex education)**		
Audio tapes	1.54	0.82
Video tapes	1.60	0.87
Books and manuals, except textbooks on sexual health	1.70	0.89
Pregnancy, HIV/AIDS, and STIs prevention materials such as posters, pamphlets, pictures	1.80	0.92
Newspapers and magazines	1.87	0.95
Textbooks	2.64	1.03
**Do you think the amount of time allocated for sexual health education in your school is adequate? (Time allocated for sex education)**	2.15	1.00

Evaluation score (4-point Likert scale, ranging from 1–4).

Out of the 21 items measuring the “information on HIV and sexual health” factor, many students reported that they received the least amount of information on “where HIV counseling and testing can be received”, with a mean value of 2.29 (SD 1.00), followed by “communication with parents or other trusted adults about sexual health topics”, with a mean value of 2.53 (SD 1.04). On the other side, the students perceived that they received the most amount of information on “susceptibility to contracting HIV/AIDS and STIs”, with a mean value of 3.33 (SD 0.85).

Out of eight items measuring the “support and involvement of teachers and parents” factor, the item that ranked the lowest according to student evaluations was “my parents/guardian participate in sexual health education”, with a mean value of 1.83 (SD 1.01). The item “teachers in this school have enough knowledge to teach sexual health education”, meanwhile, was ranked the highest, with a mean value of 2.95 (SD 0.97).

Out of the six items on the “teaching aids for sexual health education” factor, the item “audio tapes” was ranked as the least frequently used, with a mean value of 1.54 (SD 0.82). Meanwhile, the item ranked as most used was “textbooks”, with a mean value of 2.64 (SD 1.03). Finally, when students were asked if the “time allocated for sexual health education” was adequate, the mean response value was 2.15 (SD 1.00).

### Students’ attitudes toward abstinence and intentions for safer sex

Table 3 shows the results of multivariate analysis to describe the associations of students’ evaluations of school-based sex education with students’ “attitudes toward abstinence” and “intentions for safer sex.” The more “information on HIV and sexual health” received in school, the more significant was the association with more positive “attitudes toward abstinence” (β = 0.11, SE = 0.03, p = <0.018) and greater “intentions for safer sex” (β = 0.17, SE = 0.01, p = <0.001) among students. Similarly, greater “support and involvement of teachers and parents” concerning sexual health issues was also significantly associated with more positive “attitudes toward abstinence” (β = 0.16, SE = 0.06, p = <0.001) and greater “intentions for safer sex” (β = 0.15, SE = 0.03, p = <0.003) among students. Compared to male students, female students had more positive “attitudes toward abstinence” (β = 0.30, SE = 0.58, p = <0.001) as well as greater “intentions for safer sex” sex (β = 0.20, SE = 0.24, p = <0.001).

**Table 3:** School-based sex education, students’ attitudes toward abstinence, and intentions for safer sex

	**Attitudes toward abstinence **^**a**^			**Intentions for safer sex **^**b**^		
	**Beta**	**SE**	**p-value**	**Beta**	**SE**	**p-value**
**School**						
School 1 (reference)						
School 2	0.04	0.94	0.407	0.02	0.39	0.696
School 3	−0.15	1.01	**0.001**	−0.02	0.42	0.712
School 4	0.02	0.88	0.650	0.06	0.37	0.248
School 5	0.02	1.14	0.734	0.04	0.48	0.399
School 6	0.02	0.95	0.750	0.08	0.40	0.101
**Age**	−0.03	0.24	0.453	−0.01	0.10	0.811
**Gender**						
Male (reference)						
Female	0.30	0.58	**<0.001**	0.20	0.24	**<0.001**
**Grade**						
9						
10	−0.07	0.62	0.129	0.03	0.26	0.471
**Living arrangements**						
Both parents (reference)						
Single parent	−0.04	0.98	0.318	0.01	0.41	0.854
Other	−0.01	0.77	0.791	−0.02	0.32	0.673
**Father’s education**						
Illiterate (reference)						
≤Lower secondary level	0.06	0.95	0.375	0.08	0.40	0.244
≥Secondary level	−0.01	1.08	0.908	0.02	0.45	0.786
**Mother’s education**						
Illiterate (reference)						
≤Lower secondary level	0.03	0.66	0.574	0.06	0.27	0.218
≥Secondary level	0.00	1.11	0.934	0.03	0.46	0.495
**Information on sexual heath and HIV**	0.11	0.03	**0.018**	0.17	0.01	**<0.001**
**Support and involvement of teachers and parents**	0.16	0.06	**0.001**	0.15	0.03	**0.002**
**Teaching aids for sexual health education**	−0.01	0.09	0.752	−0.08	0.04	0.077
**Time allocated for sexual health education**	0.00	0.30	0.942	−0.01	0.13	0.746
**Other sources of information**	0.03	0.08	0.423	0.03	0.04	0.343

**a** = (mean = 43.18, SD = 7.12) for attitudes toward abstinence scale.

**b** = (mean = 21.82, SD = 2.92) for intentions for safer sex scale.

## Discussion

This study is the first of its kind in Nepal and, more broadly, in South Asia to show positive associations between students’ evaluations of school-based sex education and students’ attitudes toward abstinence and intentions for safer sex. Both attitudes toward abstinence and intentions for safer sex were better among students who perceived that they had received more information on HIV and sexual health through their schools. Such attitudes and intentions were also better among those students who perceived increased support and involvement in sexual health education coming from their teachers and parents. These results support the original hypothesis of our study and imply that schools, teachers, and parents may play significant roles in improving students’ attitudes and intentions regarding sexual behavior, potentially helping them to engage in responsible decision-making and eventually contributing to more positive future sexual behaviors.

In this study, the male students reported less positive attitudes toward abstinence and lower intentions for safer sex than did the female students. These trends are similar to those found in previous studies [38,39], in which measured attitudes toward sexual behavior were significantly different between male and female students.

Students evaluated the information they received on HIV and sexual health as inadequate in emphasizing life skill behaviors, emotions, relationship issues, and sexual health facilities. Owing to social and cultural taboos regarding sexual health issues in Nepal [40], many schools may have been delivering knowledge on only superficial biological facts without reference to sex or sexual relationships [23,24].

According to the students, their parents or guardians least actively participated in teaching sex education. Moreover, their parents were perceived as having low awareness of the sex education provided by the schools. This may be because Nepalese parents rarely discuss sexual health issues with their young teenage children in a family environment due to strong traditional norms and beliefs regarding sex and sexuality [41]. Additionally, they are typically less aware of their children’s education [31]. These reasons may explain the lack of parental involvement and support on sexual health issues indicated in this study.

In addition, the students evaluated that their school principals were not highly committed to providing sex education in their schools. Also, their teachers’ skills and seriousness concerning sex education were rated as limited. This is probably due to lack of specific training for teachers and health educators in Nepal [23,24]. Moreover, due to cultural factors [12,41], the teachers may harbor their own prejudices and may be reluctant to discuss sexual health issues [23,25]. They may also fear criticism by their colleagues, parents, and society at large for providing sex education [23,24]. Many teachers may lack the confidence and commitment to effectively teach sexual health topics [23,25].

Furthermore, students perceived that the teaching aids and the time allocated for sex education were insufficient. Beyond textbooks, other teaching aids for sexual health education were lacking. Several other studies have also shown a shortage of audiovisual materials to teach sex education in Nepalese schools [23,25].

Despite the significance of our findings, we should interpret them in light of several study limitations. First, we chose the schools from an urban area of Nepal through a convenience sampling method. Although this method has likely led to some sampling bias, all the schools recruited in this study follow the Nepalese government curriculum, which most of the schools across Nepal also follow. To generalize our findings, we need further studies specifically targeting rural schools to better understand the situation of school-based sex education in those areas, where HIV prevalence is high, especially in the far western region of the country [42].

Second, as the questionnaire was self-reported and included sensitive questions on sexual health issues, students might have over-or under-reported attitudes and intentions in a manner that they felt to be desirable in the Nepalese social context. However, we collected the data in schoolteachers’ absence and kept student identities anonymous to minimize any potential bias in this direction.

## Conclusions

School-based sex education in urban Nepal faces numerous challenges; our findings highlight several of the most critical gaps to be filled. Despite the challenges, our findings further disclosed that students who reported receiving more information on HIV and sexual health through their schools and whose teachers’ and parents’ showed higher levels of support and involvement in sex education had more positive attitudes toward abstinence and intentions for safer sex. These findings suggest that providing more comprehensive information on sexual health issues might improve students’ attitudes toward abstinence and intentions for safer sex. Equipping teachers with the necessary skills and optimal teaching resources may also increase the effectiveness of school-based sex education. In addition, parents’ awareness and involvement in sex education might need to be strengthened to meet students’ needs and expectations regarding sexual health issues.

## Competing interests

The authors declare that they have no competing interests.

## Authors’ contributions

RMS conceived the research questions, designed the study, conducted the fieldwork, analyzed the data, and drafted the manuscript. KO and KCP were involved in research proposal preparation, data interpretation, and manuscript revision. JY was involved in research proposal preparation and manuscript revision. ML was involved in logistic preparation for fieldwork and manuscript revision. MJ was involved in monitoring and supervising research progress. All authors critically reviewed and approved the final manuscript.

## Pre-publication history

The pre-publication history for this paper can be accessed here:

http://www.biomedcentral.com/1471-2458/13/244/prepub

## Supplementary Material

Additional file 1 **Student Questionnaire.** The student questionnaire included questions on socio-demographic characteristics of students, their perceptions about sex education in their schools, and their attitudes toward abstinence and intentions for safer sex. Click here for file
