# Supplementary material for: Better learning in schools to improve attitudes toward abstinence and intentions for safer sex among adolescents in urban Nepal
Source: BMC Public Health. 2013 Mar 20;13:244. doi: 10.1186/1471-2458-13-244 (PMC3608152; doi:10.1186/1471-2458-13-244)
Supplement: Additional file 1 — Student Questionnaire. The student questionnaire included questions on socio-demographic characteristics of students, their perceptions about sex education in their schools, and their attitudes toward abstinence and intentions for safer sex. [file 1471-2458-13-244-S1.pdf]

School code:………
Student code:………

Student Questionnaire

School-based Sex Education in Urban Schools of Nepal

This research is to help us find out about how you perceive about sex education in your school and your attitudes and intentions towards sexual behaviors. We are very grateful that you are taking part in this study and your views are very important to us.

Some questions might be sensitive and personal. However, it is very important that you give us your answer honestly and without hesitation. Please remember that there is no right or wrong answer to these questions and nobody will judge your answers. We are only interested in what people like you think and feel about the topics in the questionnaire.

Also, nobody except the researcher will see your questionnaire. Your answers will be kept confidential and we will not pass them to teachers, parents or anyone else. When we report back on the findings of our research, we will not identify you or any other individual with the answers you have given to us.

We do not force you to participate in this study. The choice is completely yours. If you choose not to answer any question, you are free to do so.

Please put a tick in the box ☑ or write an answer where asked.

S.N	Questions					
Q1.	What is your date of birth?<br>………………………….. yyyy / mm/ dd					
Q2.	What is your gender?<br>Male ☐ Female ☐					
Q3.	Which grade are you in?<br>Grade 9 ☐ Grade 10 ☐					
Q4.	Whom do you stay with?					
	Father & mother	☐	Father only	☐	Mother only	☐
	Grand parents	☐	Others (mention) ………………………..			
Q5.	What is the level of your father’s education?					
	Illiterate	☐	Can read and write	☐	Primary level (1-5)	☐
	Lower secondary level (6-8)	☐	Secondary level (9-10)	☐	Higher secondary level (11-12)	☐
	College graduate and above	☐	Vocational training	☐		
Q6.	What is the level of your mother’s education?					
	Illiterate	☐	Can read and write	☐	Primary level (1-5)	☐
	Lower secondary level (6-8)	☐	Secondary level (9-10)	☐	Higher secondary level (11-12)	☐
	College graduate and above	☐	Vocational training	☐		
Q7.	Have you ever had lessons on sexual health in your school?<br>Yes ☐ No ☐					
Q8.	In which class were you first taught about sexual health?<br>Primary level (1-5) ☐ Lower secondary level (6-8) ☐ Secondary level (9-10) ☐					
Have you learned about the following topics in your school’s sexual health education lessons?						
		Not at all	Slightly	Somewhat	Completely	
Q9.	Physical changes that take place in boys and girls during adolescence	☐	☐	☐	☐	
Q10.	Emotional changes that take place in boys and girls during adolescence	☐	☐	☐	☐	
Q11.	Relationships with opposite sex	☐	☐	☐	☐	
Q12.	Susceptibility to contracting HIV/ AIDS and STIs	☐	☐	☐	☐	

Q13.	Modes of HIV/ AIDS and STIs transmission	☐	☐	☐	☐
Q14.	Symptoms of HIV/ AIDS and STIs	☐	☐	☐	☐
Q15.	Consequences of HIV/ AIDS and STIs	☐	☐	☐	☐
Q16.	The probability of becoming pregnant or causing a pregnancy if sexually active	☐	☐	☐	☐
Q17.	Consequences of unintended pregnancy	☐	☐	☐	☐
Q18.	Abstinence	☐	☐	☐	☐
Q19.	Frequency of sex	☐	☐	☐	☐
Q20.	Number of partners	☐	☐	☐	☐
Q21.	Condom use	☐	☐	☐	☐
Q22.	STI testing and treatment	☐	☐	☐	☐
Q23.	HIV counselling and testing	☐	☐	☐	☐
Q24.	Contraceptive use, their effectiveness and how they work	☐	☐	☐	☐
Q25.	Self efficacy to refuse sex and to use condom during sexual intercourse	☐	☐	☐	☐
Q26.	Perception of peer norms about sex and perception for peer sexual behaviour	☐	☐	☐	☐
Q27.	Communication with parents or other trusted adults about sexual health topics	☐	☐	☐	☐
Q28.	Local sources for obtaining condoms and other contraceptives	☐	☐	☐	☐
Q29.	Where HIV counselling and testing can be received	☐	☐	☐	☐
Do you think that your school used following teaching materials and resources in its sexual health education lessons?					
Q30.		Not at all	Slightly	Somewhat	Completely
	Text books	☐	☐	☐	☐
Q31.	Books and manuals except text books on sexual health	☐	☐	☐	☐
Q32.	Pregnancy, HIV/ AIDS and STIs prevention materials like posters, pamphlets, pictures	☐	☐	☐	☐
Q33.	Newspapers and magazines	☐	☐	☐	☐
Q34.	Audio tapes	☐	☐	☐	☐
Q35.	Video tapes	☐	☐	☐	☐

Q36.	Religious books	☐	☐	☐	☐
Q37.	Do you think time allocated for sexual health education in this school is adequate	☐	☐	☐	☐
Do you believe in the following about your teachers and parents/ guardian?					
Q38.		Not at all	Slightly	Somewhat	Completely
	Teachers in this school have enough knowledge to teach sexual health education.	☐	☐	☐	☐
Q39.	Teachers in this school have enough skills to teach about sexual health education.	☐	☐	☐	☐
Q40.	Teachers in this school take teaching about sexual health education seriously.	☐	☐	☐	☐
Q41.	Teachers in this school are happy to teach about sexual health education.	☐	☐	☐	☐
Q42.	The Principal is committed to having sexual health education taught in this school.	☐	☐	☐	☐
Q43.	My parents/ guardian are aware about sexual health education in my school	☐	☐	☐	☐
Q44.	My parents/ guardian support sexual health education in my school	☐	☐	☐	☐
Q45.	My parents/ guardian participate in teaching sexual health education	☐	☐	☐	☐

**Attitudes Toward Abstinence**					
Q46.	It is important for me not to have sexual intercourse before I get married.				
	Strongly disagree	Disagree	Neutral	Agree	Strongly agree
	☐	☐	☐	☐	☐
Q47.	Having sexual intercourse should be viewed as just a normal and expected part of teenage dating relationships.				
	Strongly disagree	Disagree	Neutral	Agree	Strongly agree
	☐	☐	☐	☐	☐
Q48.	It is against my values for me to have sexual intercourse while I am an unmarried teenager.				
	Strongly disagree	Disagree	Neutral	Agree	Strongly agree
	☐	☐	☐	☐	☐
Q49.	A teen who has had sexual intercourse outside of marriage would be better off to stop having sexual intercourse and wait until marriage.				
	Strongly disagree	Disagree	Neutral	Agree	Strongly agree
	☐	☐	☐	☐	☐

Q50.	Teens who have been dating the same person for a long time should be willing to go along and have sexual intercourse if their partner wants to.<br>Strongly disagree ☐ Disagree ☐ Neutral ☐ Agree ☐ Strongly agree ☐
Q51.	The risk of AIDS and other sexually transmitted diseases is reason enough for teenagers to avoid sexual intercourse before they're married.<br>Strongly disagree ☐ Disagree ☐ Neutral ☐ Agree ☐ Strongly agree ☐
Q52.	It is all right for teenagers to have sexual intercourse before they're married if they are in love.<br>Strongly disagree ☐ Disagree ☐ Neutral ☐ Agree ☐ Strongly agree ☐
Q53.	Having sexual intercourse is something only married couples should do.<br>Strongly disagree ☐ Disagree ☐ Neutral ☐ Agree ☐ Strongly agree ☐
Q54.	Even if I am physically mature, that doesn't mean I'm ready to have sex.<br>Strongly disagree ☐ Disagree ☐ Neutral ☐ Agree ☐ Strongly agree ☐
Q55.	I think it is OK for kids of my age to have sex.<br>Strongly disagree ☐ Disagree ☐ Neutral ☐ Agree ☐ Strongly agree ☐
Q56.	People who do not want to have sexual intercourse should have the right to say "NO."<br>Strongly disagree ☐ Disagree ☐ Neutral ☐ Agree ☐ Strongly agree ☐
Q57.	My sexual values and beliefs agree with those of my parent(s).<br>Strongly disagree ☐ Disagree ☐ Neutral ☐ Agree ☐ Strongly agree ☐
**Intentions for safer sex**	
Q58.	I will make sure a condom is use when I have sex.<br>Agree ☐ Moderately agree ☐ Moderately disagree ☐ Disagree ☐
Q59.	I will only have one sexual relationship at a time.<br>Agree ☐ Moderately agree ☐ Moderately disagree ☐ Disagree ☐

Q60.	I do not plan on having sex until I am married.<br>Agree ☐ Moderately agree ☐ Moderately disagree ☐ Disagree ☐
Q61.	I would only have sex with a person who I have a long term relationship with.<br>Agree ☐ Moderately agree ☐ Moderately disagree ☐ Disagree ☐
Q62.	I will not have sex with someone who refuses to use a condom.<br>Agree ☐ Moderately agree ☐ Moderately disagree ☐ Disagree ☐
Q63.	I do not plan on having sex until I am at least eighteen years old.<br>Agree ☐ Moderately agree ☐ Moderately disagree ☐ Disagree ☐
